# Novel Tumor Suppressor Function of Glucocorticoid-Induced TNF Receptor GITR in Multiple Myeloma

**DOI:** 10.1371/journal.pone.0066982

**Published:** 2013-06-13

**Authors:** Yang Liu, Phong Quang, Esteban Braggio, Hai Ngo, Gayane Badalian-Very, Ludmila Flores, Yong Zhang, Antonio Sacco, Patricia Maiso, Abdel Kareem Azab, Feda Azab, Ruben Carrasco, Barrett J. Rollins, Aldo M. Roccaro, Irene M. Ghobrial

**Affiliations:** 1 Department of Medical Oncology, Dana-Farber Cancer Institute, Harvard Medical School, Boston, Massachusetts, United States of America; 2 Department of Medicine, Division of Haematology, Mayo Clinic College of Medicine, Scottsdale, Arizona, United States of America; Emory University, United States of America

## Abstract

Glucocorticoid-induced TNF receptor (GITR) plays a crucial role in modulating immune response and inflammation, however the role of GITR in human cancers is poorly understood. In this study, we demonstrated that GITR is inactivated during tumor progression in Multiple Myeloma (MM) through promoter CpG island methylation, mediating gene silencing in primary MM plasma cells and MM cell lines. Restoration of GITR expression in GITR deficient MM cells led to inhibition of MM proliferation *in vitro* and *in vivo* and induction of apoptosis. These findings were supported by the presence of induction of p21 and PUMA, two direct downstream targets of p53, together with modulation of NF-κB in GITR-overexpressing MM cells. Moreover, the unbalanced expression of GITR in clonal plasma cells correlated with MM disease progression, poor prognosis and survival. These findings provide novel insights into the pivotal role of GITR in MM pathogenesis and disease progression.

## Introduction

Tumor Necrosis Factor receptor superfamily members (TNFRSFs) play an important role in immune responses and inflammatory reactions [1–5]. It has been recently shown that TNFRSFs are also associated with the pathogenesis of Multiple Myeloma (MM) [6,7]. For example, CD40 (*TNFSF5*) mediates MM cell survival and proliferation, as well as migration through the NF-κB pathway [8]. Other members of the family such as APRIL (*TNFRSF13B*) and BAFF (*TNFRSF17*) were shown to be involved in the protection of MM cells from apoptosis via NF-κB activation [9], while loss-of-function mutations of Fas antigen (*TNFRSF6*) could inhibit Fas ligand induced apoptosis in MM cells [10]. These studies suggest that TNFRSFs could play multiple roles in the pathogenesis of MM. Glucocorticoid-induced TNFR-related gene (GITR) is a member of the TNFR super family (*TNFRSF18*) that is considered a key regulator in a multitude of immune functions. GITR is expressed and further upregulated on most immune cell types like regulatory T cells (T-regs), naïve T cells and natural killer cells (NKs) [11]. GITR plays a pivotal role in inflammation processes and autoimmune diseases. It is triggered by its ligand (GITRL), mainly expressed in antigen-presenting cells and endothelial cells [12]. Conversely, GITR engagement in NK cells induces an inhibitory effect, even though a separate study provides opposite results, demonstrating that co-stimulation by GITR/GITRL interaction is found either to activate or to inhibit NK cells [13,14]. GITR expression in tumor infiltrating lymphocytes has been found to be associated with cancer progression in patients suffering from esophageal adenocarcinomas. However, the role of GITR as a direct regulator of tumor progression in MM has not been previously described.

Multistep tumor progression occurs as a succession of clonal expansions, which is triggered by acquisition of an enabling mutant genotype. Clonal expansion may occur due to acquired mutations or epigenetic alterations such as DNA methylation and histone modifications affecting the regulation of gene expression. In general, cancers are characterized by a global DNA hypomethylation and locus-specific hypermethylation of tumor suppressor genes.

In this study, we demonstrated that GITR is inactivated during tumor progression in MM through promoter CpG island methylation, mediating gene silencing in primary MM plasma cells and MM cell lines. Restoration of GITR expression in GITR deficient MM cells led to inhibition of MM proliferation *in vitro* and *in vivo* and induction of apoptosis. Notably, GITR/GITRL interaction increased the level of p53-regulated genes, such as *CDKN1A* (p21) and *BBC3* (PUMA) in a ligand dependent manner. Mechanistically, we demonstrated that GITR negatively regulates the NF-κB signaling pathway in MM cells leading to apoptosis in response to TNF-α. These findings imply that GITR acts as a potential tumor suppressor gene in MM, and its epigenetic silencing facilitates NF-κB activation and tumor proliferation in MM.

## Materials and Methods

### Ethics statement

Bone marrow samples from patients with MM were obtained under Dana-Farber Cancer Institute IRB approval with written informed consent and according to the declaration of Helsinki. In *in vivo* studies, mice were treated, monitored, and sacrificed in accordance with approved protocol of the Dana-Farber Cancer Institute Animal Care and Use Committee.

### Cultured cell lines and primary tumor samples

Five human myeloma cell lines were used: MM1.S, U266, RPMI (ATCC, Manassas, VA); OPM1 and INA6 (kind gift of Dr. K. Anderson, Dana-Farber Cancer Institute, Boston, MA) (15). Cells were cultured in RPMI1640 medium with 10% FBS. The umbilical vein endothelial HUVEC cell line (Cambrex, Walkersville, MD) was cultured in EGM-2 MV media (Cambrex) reconstituted according to the manufacturer. Plasma cells from patients with multiple myeloma were obtained using anti-human CD138 microbead selection (Miltenyi Biotec, Auburn, CA). GFP+/GITR+ MM.1S cells were generated using lentivirus based transfection approach.

### Methylated DNA immunoprecipitation (MeDIP) assay

25 mg of gDNA sample were diluted in TE buffer (10 mMTris-HCl, pH 7.5, 1 m MEDTA) and sheared to between 300 to 800 bp. 4 µg DNA of each sample was saved as input and the rest heated to 95°C for 10 min and immediately placed on ice. Immunoprecipitation was performed using 10 µg anti-5MeCyt monoclonal antibody for (Eurogentec, Bi-MECY-0100) sheared gDNA in IP buffer (20 mM Na-Phosphate, pH 7.0, 1 M NaCl, 2% Triton-X100). MeDIP was purified and precipitated using phenol and chloroform: isoamyl alcohol. The extent of methylated DNA enrichment in our MeDIP samples was verified by qPCR on an Applied Biosystems 7500 Real-Time PCR System using the following primers:

TNFRSF18F: 5’-TTCAAGAGCCCACAGCCAGTTG-3’

TNFRSF18R: 5’-ATTCTCAGGTCATGAACGGTCCC-3’.

TNFRSF11AF: 5’-GAGTTTTGGGGAGCGGAGGC-3’

TNFRSF11AR: 5’-GTTATCTGCCGCAAGTGAGG-3’

TNFRSF9F: 5’- CATTGAGCAGCTGGAGAACCTC-3’

TNFRSF9R: 5’-CGTCCGCATCTGTCCGCATC-3’

TNFRSF19F: 5’- CACTCATCCGCTTCAGTAGCC -3’

TNFRSF19R: 5’- GCACACCGTGCACCTCCCAG-3’

TNFRSF21F: 5’-GAGAGGTCCCCATGGCTGAAC-3

TNFRSF21R: 5’- GTGCTGAGCGCCCCTAGAGC-3’

TNFRSF8F: 5’- GCTGGGACTGATTCATTCATTC-3’

TNFRSF8R: 5’- CGGACGACCAACCTCCCATG-3’

TNFRSF11BF: 5’- CTTACCACGAGCGCGCAGC-3’

TNFRSF11BR: 5’-CTGATCAAAGGCAGGCGATAC-3’

The relative changes in the extent of promoter methylation were determined by measuring the amount of promoter in immunoprecipitated DNA after normalization to the input DNA %(MeDNA-IP/Input) = 2^[(Ct(input)-Ct(MeDNA-IP) ×100.

#### Bisulfite sequencing and methylation specific PCR

Genomic DNA was prepared and bisulfite converted by EpiTect Plus DNA Bisulfite Kit (Qiagen, Valencia, CA). PCR products were purified and cloned into PGEM-T-easy vector (Promega, San Luis Obispo, CA), then sent for DNA automatic sequencing. Primer sequences for GITR are Forward 5’-GGGTTATTGGTTGGAGTTGGTAT-3’, reverse 5’-CCTATCCCCATTCTATAAAATTCC-3’. Primer sequences for MSP are

MF: 5’- GCGGCAGCAGCGCGTCGTGTGAACCCG-3’,

MR: 5’- CGCGTTTCGAACCCTATACGA-3’

UF: 5’- GTGGTAGTAGTGTGTTGTGTGAACCTG-3’

UR: 5’- CACATTTCAGGCCCTGTGCAG-3’

#### Bone marrow Tissue Immunohistochemistry

Bone marrow tissues were obtained from both healthy individuals and MM patients. Samples were embedded in paraffin-decalcified in Rapid-Cal-Immuno (BBC, Stanwood, WA) for 1 hour, washed, and then paraffin embedded. For GITR staining, 3- to 4-μm tissue sections were mounted on plus slides, dried for 2 hours in a 60°C oven, and then stained using GITR polyclonal antibody (R&D system, Minneapolis, MN, cat# AF689; 1:200 dilution) according to established protocols in a BenchMark automated immunostainer (Ventana Medical Systems, Tucson, AZ). Heat-induced epitope retrieval was performed with the EDTA-buffered cell conditioning (CC1) retrieval solution. Immunostaining was completed with the iView diaminobenzidine tetrahydrochloride (DAB) detection kit (Ventana). According to the description of GITR antibody, immersion fixed paraffin-embedded sections of human tonsil was subjected to GITR antibody and considered as positive control.

#### Lentiviral infection

PCDH-ef1-CMV-EGFP lentiviral vector was purchased from SBI Company. cDNA clone of GITR was purchased from Origene company. cDNA of GITR was amplified and subcloned into PCDH-ef1-CMV-EGFP using BamHI and XbaI restriction enzyme sits. The PCDH-empty control and PCDH-GITR plasmid were co-transfected with pPACK™ packaging plasmid mix (SBI, LV050A-1) into 293T cells, which is available in the frozen stock of the lab. Viral supernatant was obtained after 72 hrs post-transfection. MM1.S and OPM1 cell lines were infected by PCDH-control and PCDH-GITR recombinant lentivirus for 72 hours. Both GFP positive and GFP negative MM1.S and OPM1 were sorted by FACS.

#### Competition assay and cell proliferation assay

The GFP competition assay was used to assess the ability of proliferation of two days after infection. MM cells were split into replicate wells of 500,000 cells in 12-well plates. After 24-h treatments with a range of drug doses, the GFP-positive percentage was quantified in the surviving cell population by using a BD Biosciences LSRII flow cytometry. GFP+ and GFP-cells were sorted and combined as 1:1 ratio. The percentage of GFP+ cells was examined everyday by flow cytometry. Proliferation rate and cytotoxicity were measured by DNA synthesis using BrdU (R&D) assay and by 3-(4,5-dimethylthiazol-2-yl)-2,5 diphenyltetrazolium bromide (MTT; Chemicon International, Temecula, CA) dye absorbance, respectively.

#### Cell cycle analysis

For cell cycle analysis by flow cytometry, cells were seeded in a 10 cm dish at 60% confluency. The cells were concomitantly treated with RNase A (Sigma Aldrich, San Luis, MO) and stained with 10ng/ml propidium iodide. Cell cycle status was determined using a FACS caliber flow cytometer (Becton Dickinson, Oxford, UK) and analyzed using FlowJo7.6.5 software.

#### Immunoblotting

Whole-cell lysates were subjected to sodium dodecyl sulfate-polyacrylamide gel electrophoresis (SDS-PAGE) and transferred to polyvinyldene fluoride (PVDF) membrane (Bio-Rad Laboratories, Hercules, CA). The antibodies used for immunoblotting included anti–caspase3, p-Ikk-β, Ikk-β, IκB, P65, P50, (Cell Signaling Technology, Danvers, MA), -p53 and actin (Santa Cruz Biotechnology, Santa Cruz, CA). Nuclear extracts of the cells were prepared using the Nuclear Extraction Kit (Panomics, Redwood City, CA) and subjected to immunoblotting with anti-p65, -p50/p105, (Cell Signaling Technology, CA), and anti-nucleolin (Santa Cruz Biotechnology, CA) antibodies. In addition, the GITR antibody (R&D Cell Biology, CA) was used for immunoblotting and immunohistochemistry on tissue microarray sections.

#### Active Motif for NF-κB activity

NF-κB activity was investigated using the Active Motif TransAM NF-κB Family Kit, a DNA-binding enzyme-linked immunosorbent assay (ELISA)-based assay (Active Motif, North America, Carlsbad, CA). Briefly, MM.1S cells (empty control MM1.S and GITR-transfected MM1.S) were cultured in presence or absence of TNF-α (10ng/ml) for 20 minutes. NFκB-p65, -p50 transcription factors binding to the related consensus sequence on the plate-bound oligonucleotide were studied from nuclear extracts, following the manufacturer’s procedure.

#### In vivo tumor progression of MM cells

Seven SCID mice for each group were injected with 5X10^6^ MM1.S cells. After 4 weeks of tail vein injection, the bone marrow from the femur and skull, as well as peripheral blood were isolated from the two groups of mice. Mice bone marrow tissues were crushed and filtered into single cell suspension. Anti-human CD138 monoclonal antibody (BD bioscience, San Jose, CA) as used to examine MM cells growth by both flow cytometry and immunofluorescence.

#### Immunofluorescence

Immunocytochemical analysis was performed using an epifluorescence microscope (Nikon Eclipse E800; Nikon, Avon, MA) and a Photometrics, Cool snap CF color camera (Nikon, Lewisville, TX). Briefly cells were fixed with 4% paraformaldehyde in phosphate-buffered saline and permeabilized with 0.2% Triton X-100. Cells were blocked in 1X PBS / 5% normal goat serum PBS and incubated with primary antibody at 4°C overnight. Cells were incubated with Alexa Fluor 488 goat anti-rabbit secondary antibody for 2hrs.

#### cDNA expression profiling

Total RNA of control_MM1.S and GITR_MM1.S was isolated using an RNeasy kit (QIAGEN, Valencia, CA). Quality control of RNA was done using RNA6000 Nano assay on the Agilent 2100 Bioanalyzer (Santa Clara, CA). Hybridization to the Human U133A 2.0 microarray was performed in Dana-Farber Cancer Institute core facility. In the experiment comparing the overexpressing GITR versus empty control, the list of filtered probe-set was analyzed looking for differentially expressed genes between experiments (>2-fold change).

#### Analysis of expression profiling of available datasets

*GITR* expression was analyzed in publicly available datasets comprising bone marrow plasma cells from healthy donors (NPC) and different stages of the MM disease. NPC, MG, US, SMM data was obtained from GEO dataset GSE5900and MM data from GSE2658 and the MMRC portal (data available at www.broadinstitute.org/mmgp/home). All the intensity files were MAS5 transformed and the data was normalized to the median. Comparisons between two groups were performed using t-test (2 tailed) and for more than 2 groups using ANOVA. The comparison of *GITR* expression level between molecular MM subgroups was performed using the two most relevant molecular classifications in MM: the TC and the UAMS classifications (16,17). Results were considered significant when p<0.05.

#### Statistical Analysis

Statistical significance of differences in the different groups was determined using Student’s *t*-test. The minimal level of significance was p<0.05. Experiments were repeated in triplicates. Error bars reported in the figures represent standard deviations.

## Results

### GITR is aberrantly methylated in both primary MM cells and MM cell lines

**Figure 1:**
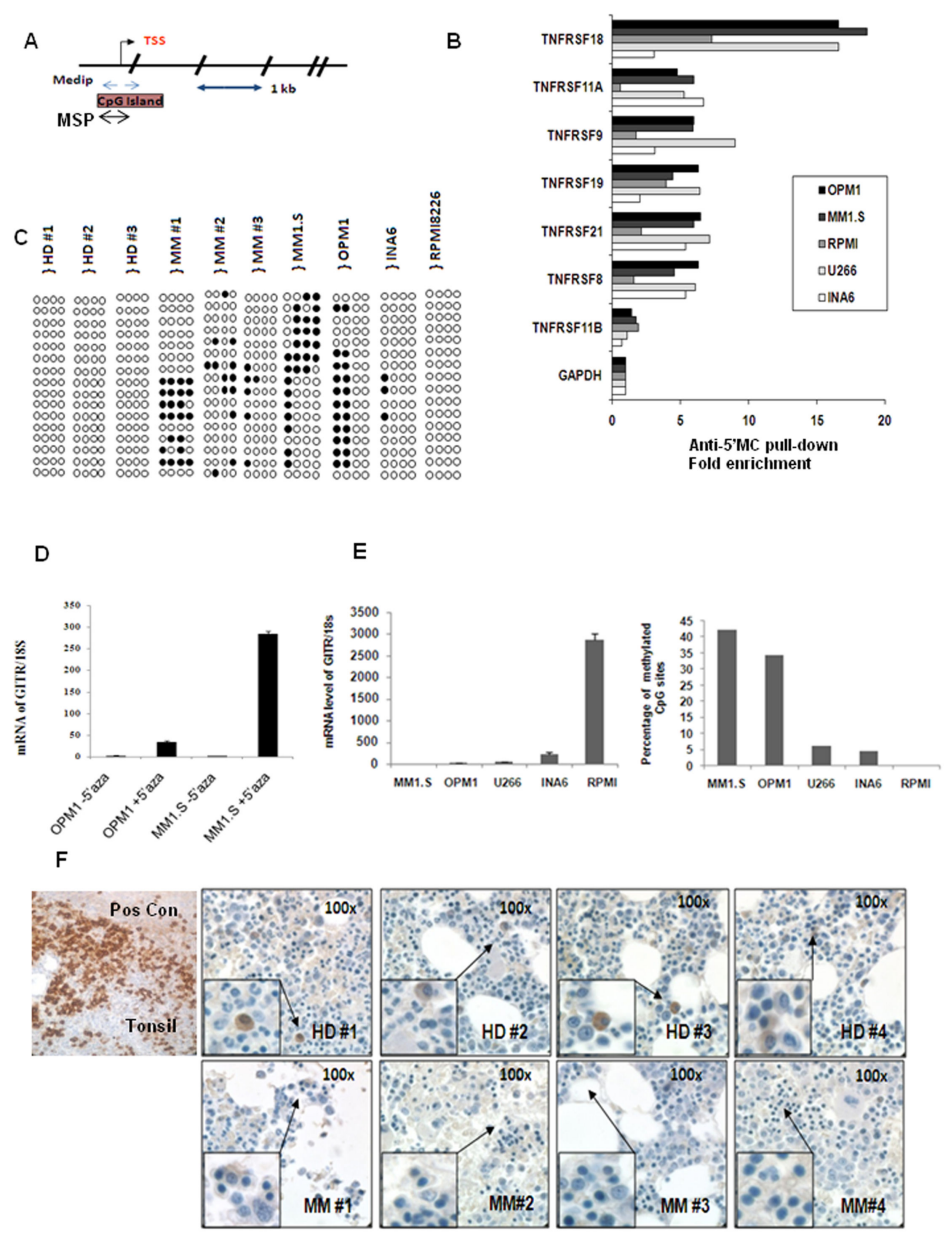
Promoter CpG island Hypermethylation leads to repression of GITR in MM cells. A) Primer location for Medip, MSP and bisulfite sequencing assay. Bar arrow represents 1 κB region on the genome. TSS represents transcription starting site. B) Analysis of DNA methylation status of 7 TNFRSF members promoter CpG islands in 5 MM cell lines. Methylated DNAimmunoprecipitation assay was performed to profile promoter methylation status. Promoter of housekeeping gene GAPDH was used as unmethylated control. Fold enrichment was calculated as described in the method and normalized to GAPDH. C) Bisulfite sequencing at the CpG island of GITR promoter, with circles denoting the methylation status of each selected clone. Black and white circles are methylated CG dinucleotides, and non-methylated CpG dinucleotides and TG dinucleotides, respectively. D) Re-expression of GITR mRNA level was examined by real-time PCR in the presence of 5’ azacytidine (5 µM) for 4 days. Total RNA was extracted and reverse transcripted with oligo_dT. The mRNA level of GITR was normalized to 18s. Mean±SD. E) Expression of GITR was determined in 5 MM cell lines by real-time PCR. The mRNA level was normalized to 18s. Mean±SD. DNA methylation status of GITR in 5 MM cell lines was calculated by counting the methylated CG sites in the promoter of GITR and divided by total CG number. F) Expression of GITR examined in 9 primary MM and 11 normal bone marrow by tissue array using anti-human GITR monoclonal antibody. 4 slides of each group were shown. GITR expression was represented as brown color. Staining of tonsil for GITR staining was considered as positive control.

We hypothesized that deregulation of TNFR super family members may play a pivotal role in modulating MM pathogenesis. We therefore evaluated the expression of TNFRSFs in primary MM cells by analyzing GEO dataset GSE2658 and found that 7 members of the TNFRSF family including*TNFFSF18* (GITR), *TNFRSF11A*, *TNFRSF9*, *TNFRSF19*, *TNFRSF8*, *TNFRSF21* and *TNFRSF11B* exhibited lower expression levels in MM cells compared to their normal cellular counterpart (Figure S1a). We next investigated the mechanisms responsible for down-regulation of TNFRSFs members; and profiled DNA methylation status of the promoter CpG islands (CGI) of the related genes using MeDIP assay in 5 MM cell lines. Primers were designed within 500bp region around the CpG islands at the promoter region (Figure 1a). MM cells presented with significant methylation in the promoter CGI of GITR (TNFRSF18), with specific higher methylation in OPM1, MM.1S and U266 cells, compared to RPMI.8226 and INA6, where lower methylation levels were documented (Figure 1b). We further confirmed that hypermethylation of GITR promoter occurred in MM cells, by performing MeDIP assay using MM1.S and OPM1 cell lines treated with the demethylating agent, 5’ azacytidine. Untreated cells were used as control, (Figure S2a).

To further evaluate the methylation status of GITR in MM cells, we performed methylation-specific PCR (MSP) for GITR in MM cell line and primary MM bone marrow CD138+ plasma cells (Figure S2b). The MSP result was further validated by bisulfate conversion. All the identified methylated CpG sites were represented as dark circles, whereas unmethylated sites in that same region were represented as blank circles across the entire CpG island (Figure 1.c). Further confirmation was performed by sanger sequencing (Figure S2.c). Based on the results of MSP and bisulfate sequencing, we found that primary clonal plasma cells presented with promoter GITR methylation in 3 out of the 5 MM patients studied, compared to the related normal cellular counterpart, where an unmethylated status was observed. Notably, primary MM cells and MM cell lines presented with a similar methylation pattern: specifically, the hypermethylation was mainly demonstrated within the first 130 base pairs of CpG island located at the promoter region In addition, we found that expression of GITR negatively correlated with the promoter CGI methylation status, indicating that loss of GITR expression correlates with aberrant DNA methylation in MM cells (Figure 1e). These observation were further confirmed by exposing MM cells to 5’ azacytidine treatment that led to increase of GITR levels, both in MM.1S and OPM1 cells (Figure 1d), while no relatively significant increase of GITR expression was obtained by performing qPCR in INA6, U266 and RPMI8226 cell lines, which were characterized by lower methylation pattern of GITR promoter (Figure S1d). Furthermore, these data were also confirmed at protein level; GITR expression significantly increased upon 5’ azacytidine treatment in a dose dependent manner, as detected by flow cytometry (Figure S3b). These findings suggest that deregulation of GITR may result from an aberrant promoter CGI methylation in MM cells.

The expression of GITR was next evaluated using immunohistochemistry in bone marrow specimens of both MM patients and healthy individuals 82% of the normal bone marrows evaluated showed GITR positivity, compared to MM specimens that were GITR positive only in 33% of the cases (Figure 1e).

### Deregulation of GITR correlates with MM progression, prognosis and survival

**Figure 2:**
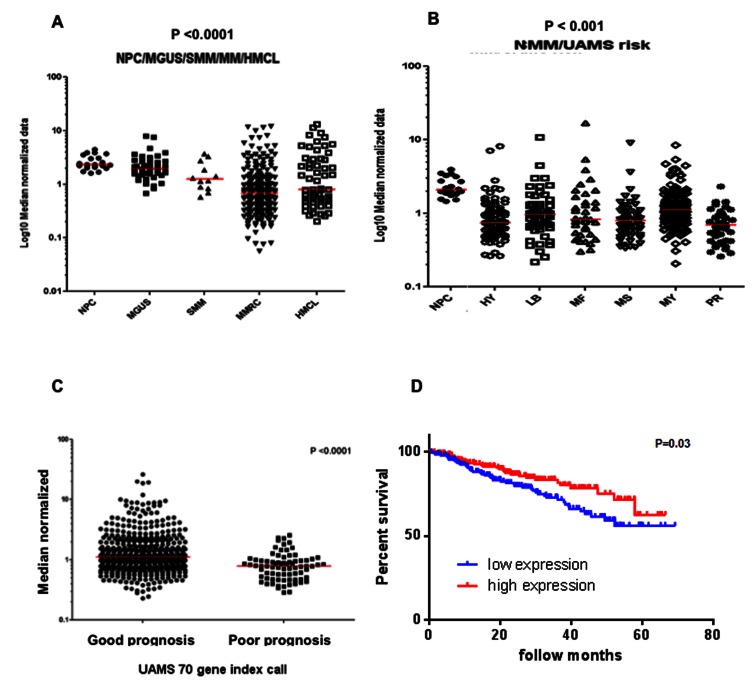
Expression of GITR correlates with MM clinical pathogenesis. A) Expression of GITR was evaluated by Gene expression profiling analysis (GEO 2658 datestes). The average of GITR expression in MG, US and smoldering MM was labeled as red bar. B) Expression of GITR was evaluated by Gene expression profiling analysis (GEO 2658 datestes). The average of GITR expression in every MM subtype was labeled as red bar. >C) Patients belonging to Total Therapy 2 (TT2) and Total Therapy 3 (TT3) trials with available gene expression data were divided in poor and good prognosis groups based on the 70-gene index generated by the University of Arkansas for Medical Sciences (UAMS). The cutoffs for the poor prognosis groups were 0.66 and 0.79 for TT2 and TT3, respectively. Median normalized TNFRSF18 expression level was compared between good and poor prognosis groups. D) Kaplan-Meier curve showing survival of 414 MM patients in GITR high and GITR low groups. The two groups were divided by the median of GITR expression in GEP datasets (GSE2658 and GSE5900) and analyzed by graphpad software (P=0.03).

We next evaluated the GITR mRNA expression levels in MM patients-derived CDC138 cells, using publically available gene expression data sets (GSE5900; GSE2658); and found a significant reduction of GITR expression in MM samples, compared to healthy individuals, and interestingly, we found a sustained reduced expression of GITR with disease progression, as shown by comparing MG, US to smoldering MM to active symptomatic MM patients (Figure 2a; P<0.001). Further expression analysis was performed in the subclasses of MM patients, showing that the lowest expression of GITR was observed in PR group, which has an advanced and more proliferative form of myeloma (Fig.2bP < 0.01). We next evaluated the expression of GITR according to prognosis and survival; and found that lower expression of GITR correlated with poor prognosis and survival in MM patients, further suggesting the functional role of GITR in modulating MM pathogenesis and disease progression (Figure 2c, P<0.001; Figure 2d, P 0.03).

### Functional role of GITR in MM cells

**Figure 3:**
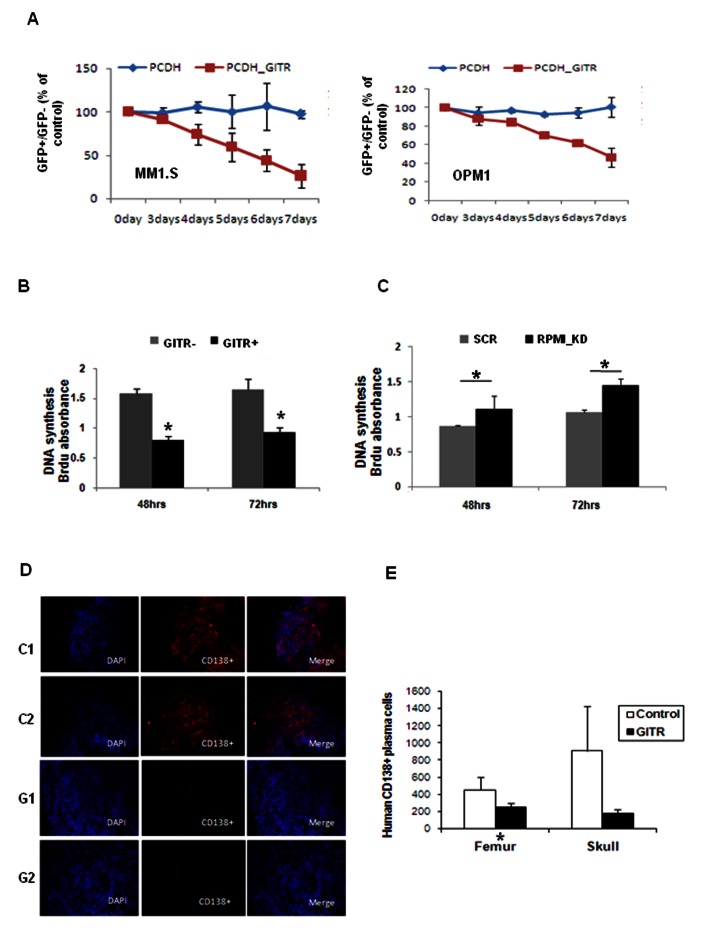
Effect of GITR on MM tumor proliferation in vitro and in vivo. A) MM cells (MM.1S and OPM1) were transfected with PCDH empty and PCDH-GITR with GFP labeled lentiviral vector (Cat# CD511B-1, SBI Inc.) respectively. Cell proliferation has been evaluated by using GFP competition assay. Expression of GFP and GITR has been examined by flow cytometry by using anti-human GITR PE labeled primary antobidy. GFP+ and GFP-MM cell were sorted by BD laser II flow machine. Ratio of GFP+/GFP-was recorded everyday after combination. B) Effect of GITR-overexpression on MM1.S cell line proliferation. 96 wells plate coated with MM1.S cells were read after 48 and 72 hours respectively. Anti-Brdu-pod was used to detect the absorbance. Mean±SD. *, P<0.05. C) Effect GITR-knockdown on RPMI.8226 cell line proliferation (RPMI.8226). 96 wells plate coated with MM1.S cells were read after 48 and 72 hours respectively. Anti-Brdu-pod was used to detect the absorbance. Mean±SD. *, P<0.05. D) SCID mice were injected i.v. with 5 million MM1.S cells, transfected with either empty vector (contrl C1, C2) or GITR (GITR+, G1, G2). In vivo tumor progression has been evaluated by using immunofluorescence staining with anti-human CD138 monoclonal antibody after 4 weeks injection, on bone marrow femurs. E) Detection of MM cells from tissues of mice injected with either empty vector (control) or GITR (GITR+). MM cells have been detected by using flow cytometry analysis for CD138. Mean±SD. *, P<0.05; **, P<0.01.

We next dissected the functional relevance of GITR expression in modulating MM cell behavior, both *in vitro* and *in vivo*. We performed GFP competition assay by introducing lentivirus-based vector with GFP labeling into MM1.S and OPM1 cell lines. Both lenti-CMV-GFP and lenti-GITR-GFP were selected by flow cytometry and combined with GFP negative MM cells in 1:1 ratio. Expression of GFP was measured by flow cytometry (Figure S4a). We found that the GITR+/GFP+ cells exhibited a competitive disadvantage compared to GITR-/GFP+ cells in MM1.S, OPM1 cell lines, indicating that expression of GITR could lead to increased cytotoxicity of MM cells (Figure 3a). Cell proliferation was next evaluated in GITR expressing MM1.S cells (GITR+), compared to control cells (GITR-); we showed that inhibition of proliferation was observed in GITR-overexpressing cells (Figure 3b). We next evaluated how GITR loss of function would affect proliferation of MM cells that present with higher GITR levels, such as RPMI8226 cells; and demonstrated that reduced expression of GITR in RMPI82226 cells led to increased cell proliferation (Figure 3c). GITR knockdown efficiency in RPMI8226 cells has been determined by real-time PCR (Figure S4b). Due to the importance of neo-angiogenesis to support MM disease progression and due to the presence of GITR specific ligand (GITRL) [15] on the surface of endothelial cells (HUVECs), we next investigated the role of GITR in MM cells in the presence of endothelial cells. *In vitro* co-culture of GITR-overexpressing MM1.S with HUVECs led to significant inhibition of cell growth, thus providing a further evidence of the tumor suppressor role of GITR in MM (Figure S4c).

We next validated our *in vitro* findings by using an *in vivo* xenograft MM mouse model. GITR expression was enhanced in MM1.S cells by using lentiviral vector. 5x10^6^ GITR+ MM1.S cells and GITR-empty vector infected control MM1.S cell were injected into SCID-bg mice via tail vein respectively. After 4 weeks, bone marrow tissues were extracted from both control and experimental mice groups. We observed significant reduction of growth of MM cells in mice that received GITR-overexpressing cells, compared to control groups, suggesting that GITR is a critical regulator of tumor growth in MM cells both *in vitro* and *in vivo* (Figure 3d-3e). Taken together, these studies suggest that GITR acts as a potential tumor suppressor in MM.

### GITR induces expression of p21 and PUMA in a ligand dependent manner

**Figure 4:**
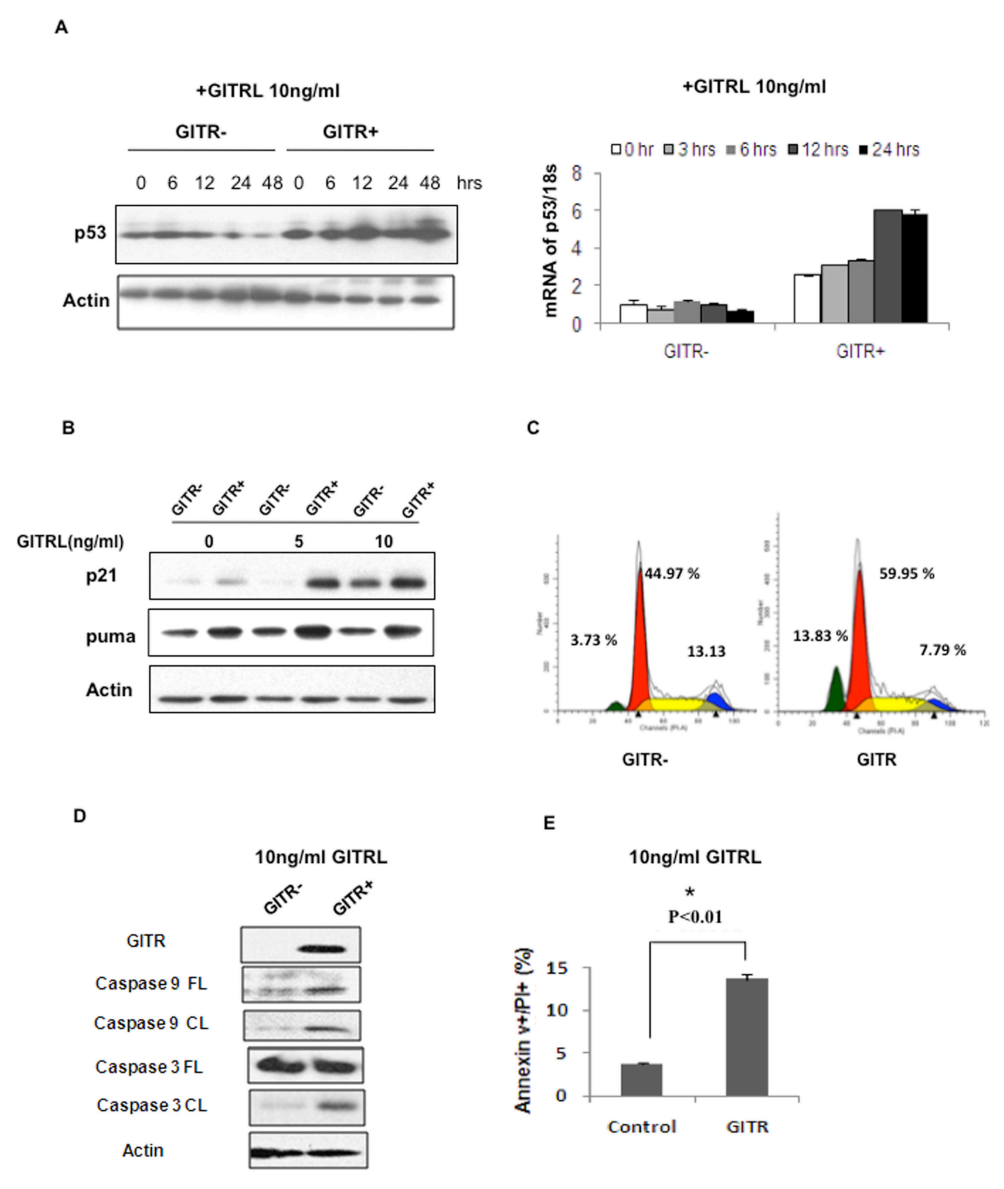
GITR induces up-regulation of p21 and PUMA in MM cells. A) Total protein and RNA was extracted in 0, 3, 6, 12, 24 and 48 hours after treatment with GITRL (10ng/mL) from GITR- and GITR+ MM1.S cells. Both Protein and mRNA level of p53 have been evaluated by western blot and qRT-PCR. Actin was considered as the protein loading control. CT value of qRT-PCR was normalization to 18s. Mean ±SD. B) GITR- and GITR+ cells were exposed to GITR-L (0-5-10ng/mL) for 24 hrs. whole cellular protein was extracted and subjected to Western blot using anti-p21, -PUMA and -actin antibodies. C) GITR- and GITR+ MM1.S cells were exposed to GITR-L (5-10 ng/mL) for 24 hours respectively. Cell cycle has been evaluated by using PI staining and flow cytometry analysis. D) GITR- and GITR+ MM1.S cells were exposed to GITR-L (5-10ng/mL) for 24 hours. Whole protein lysates have been subjected to Western blot using anti-caspase-3, 9 and –tubuilin antibodies. E) GITR- and GITR+ MM1.S cells were exposed to GITR-L (5-10ng/mL) for 24 hours. Cells were stained by PI/Annexin V to evaluate apoptosis effect using flow cytometry. Mean ±SD.

To understand the molecular basis of the role of GITR as a potential tumor suppressor in MM, we analyzed the specific gene expression signature in GITR overexpressing cells compared to cells transfected with empty vector. Gene annotation analysis showed that the p53 signaling pathway represents the most significantly enriched gene set, (P<0.001), together with up-regulation of MDM2, CDKN1a (p21), E2F1, Fas, IGF-BP3 and CD82 genes (Table. S1). Meanwhile, apoptosis response genes including Tumor Necrosis Factor Receptor Type 1-Associated Death Domain protein (TRADD) and Death Associated Protein (DAP) were also upregulated in GITR-overexpressing MM cells. Expression of these genes was validated in both GITR+ MM1.S and GITR knockdown RPMI8226 cells by real time PCR; showing overexpression of GITR could increase the level of p21, Fas, TRADD and DAP, in contrast knockdown of GITR could lead to decreased mRNA level of these genes (Figure S5a). Based on these results, we examined the expression of p53 in MM cells in response to stimulation with GITRL using qRT-PCR and western blot. We found that expression of GITR could lead to both increased mRNA and protein level of p53 in 12hrs after stimulation with 10ng/ml GITRL (Figure 4a). The elevated p53 protein could subsequently lead to the induction of p21, which is another downstream target of p53 and a regulator of mitochondrial-mediated apoptosis (Figure 4b, and Figure S5b). Next, we evaluated whether elevated levels of p21and puma proteins could affect MM cell cycle progression or apoptosis. We demonstrated that GITR expressing cells presented with increased numbers of cells in subG1/G1 phase, together with S and G2 phase inhibition (Figure 4c). Induction of apoptosis by GITR expression was also confirmed using flow cytometry with PI/Annexin V dual staining combined with immunoblotting for caspase cleavage in the presence of GITRL (Figure 4d-e). Our results indicate that GITR could modulate p53-mediated p21 and puma expression, thus mediating cell cycle progression and apoptosis in MM cells.

### GITR negatively regulates NF-κB pathway in MM cells

**Figure 5:**
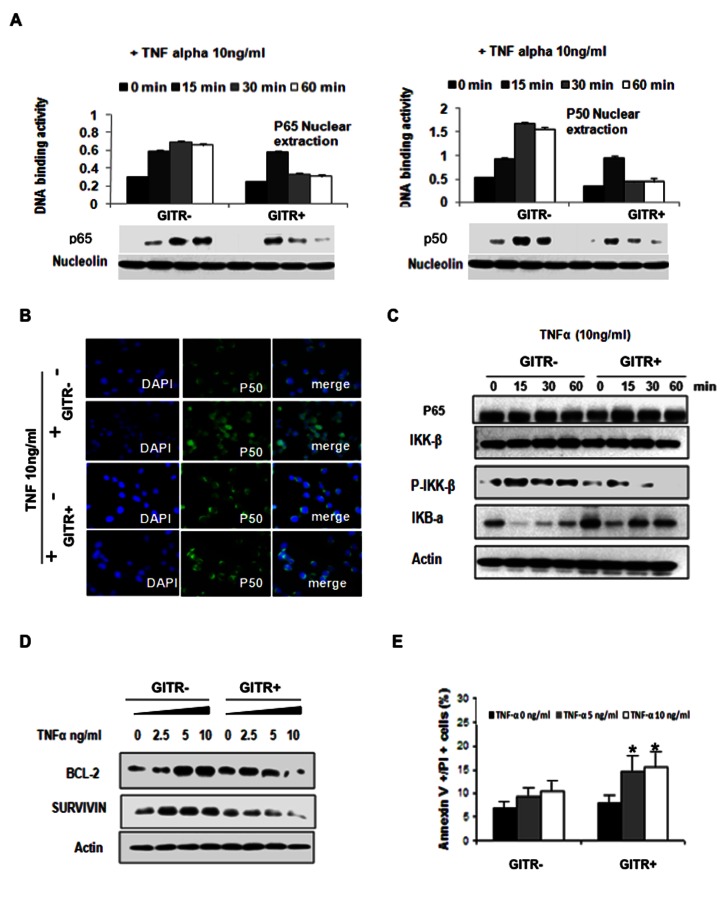
Effect of GITR on canonical NF-κB pathway. A) Effect of GITR on TNF-α induced NF-κB activation. NF-κB activity has been evaluated by using DNA binding ELISA assay in GITR- and GITR+ MM1.S cells. Cells were exposed to TNF-α (10ng/mL) for 15-30-60 minutes. NF-κB p65 and p50 transcription factor binding to its consensus sequence on the plate-bound oligo nucleotide was examined from nuclear extracts. Data represent mean plus or minus SD of triplicate experiments. Nuclear protein lysates were also subjected to Western blot using anti-p50, -p65 and -nucleolin antibodies. B) GITR- and GITR+ were harvested at 24 hours after treatment with and without TNF-α (10 ng/mL) for 60 minutes; Immunocytochemical analysis was assessed using anti-phospho-NF-κB-p50 antibody, with DAPI used to stain nuclei. C) GITR- and GITR+ cells were exposed to TNF-α (10ng/mL) for 15-30-60 minutes. Whole cellular protein lysates have been subjected to Western blot using anti-p65, -phospho(p)-IκB beta, -IκB beta, -IκB alpha, and -actin antibodies. D) GITR- and GITR+ cells were exposed to TNF-α (2.5-5-10ng/mL) for 16 hours. Whole cellular protein lysates have been subjected to Western blot using anti-BCL-2, -survivin and -actin antibodies. E) GITR- and GITR+ cells were exposed to TNF-α (2.5-10ng/mL) for 12 hours. Apoptosis was evaluated by Annexin/PI staining and flow cytometry analysis. Mean±SD.

NF-κB transcription factors play a key role in the survival and proliferation of several hematologic malignancies, including MM [16]. It has been shown that GITR may negatively regulate NK cell activation through suppression of the NF-κB pathway [13]. We therefore dissected the role of GITR in modulating the canonical NF-κB pathway in both GITR- and GITR+ cell lines. Cells were exposed to TNF-α (10ng/mL) and nuclear DNA binding activity and p50/p65 units was studied by using DNA binding ELISA assay and immunoblot at 0,15, 30 and 60 minutes. We showed that TNF-α was able to activate NF-κB pathway in both control and GITR expressing groups at 15 min (Figure 5a). However, NF-κB activation was abolished at early 30 to 60 min in GITR+ cells after the stimulation, whereas the NF-κB signal remained elevated up to 60 min in the GITR-cells, suggesting a role of GITR in terminating TNF-α-induced NF-κB activation (Figure 5a). In addition, we also demonstrated inhibition of TNF-α-dependent p50 nuclear translocation in GITR+ cells, as shown by immunofluorescence (Figure 5b). Previous studies showed that IKK-β represents a critical upstream regulator of P65/p50 activation [17]; we therefore examined the phosphorylation of IKK-β stimulated by TNF-α by immunoblotting. Consistently, the phosphorylation of IKK-β was markedly diminished by the increased expression of GITR at early time points. IΚB-α-degradation was also measured in TNF-α stimulated control and GITR overexpressing cells. We found that IΚB-α protein decreased back to the basal level at 30 to 60 min time points in GITR expressing MM1.S cells, suggesting that TNF-α stimulated NF-κB activation is IΚB-α-dependent (Figure 5c).

To further confirm that GITR induces inhibition of TNF-α-induced NF-κB signaling, we evaluated the expression p65/p50-downstream targets, such as BCL-2 and survivin. As demonstrated at protein level, BCL2 and survivin expression was upregulated in GITR-MM cells, upon stimulation with TNF-α. In contrast, no significant changes of BCL-2 and survivin were observed in GITR overexpressing cells in response to TNF-α (Figure 5d).

Previous studies showed that TNF-α alone may be responsible for NF-κB nuclear translocation, cIAP-1 and cIAP-2 up-regulation, thus leading to increase in MM cell proliferation [18]. To explore the effect of GITR on apoptosis induced by TNF-α in MM cells, we performed PI/Annexin dual staining assay. We found that GITR was able to counteract NF-κB mediated anti-apoptosis signals and facilitate the apoptosis induced by TNF-α in GITR expressing MM cells (Figure 5e). Taken together, these findings further support our hypothesis that GITR negatively regulates canonical NF-κB pathway activation by inhibiting phosphorylation of IKK-β in MM cells at early time points.

## Discussion

MM is a plasma cell neoplasia characterized by activation of the NF-κB pathway through cell autonomous mutations or external signaling from the tumor microenvironment [19,20]. In this study, we demonstrated that epigenetic silencing of *GITR* is involved in MM cell proliferation, is associated with an anti-apoptosis effect and is contributing to NF-κB-mediated MM pathogenesis. Notably, using bisulfite sequencing, we observed that all of the methylated cytosines are distributed at the first 130 base pair of promoter CGI region in both MM cell lines and primary bone marrow CD138+ MM cells, suggesting that the aberrant methylation of GITR promoter in MM cells is not a random epigenetic event. To link the function of GITR to MM cell behavior, we sought to investigate if the expression of GITR correlates with MM cells proliferation. Indeed, we demonstrated that expression of GITR could inhibit MM cells proliferation in vitro and in vivo. These results indicate that GITR acts as a potential tumor suppressor gene on MM cells and we confirmed that its re-expression induces apoptosis in MM cells. These results are consistent with previous reports indicating that deletion of GITR could lead to increased proliferation in T lymphocytes [21].

GITR is located on chromosome 1p36. Deletion of 1p36 is associated with increased risk for neoplasia, including neuroblastoma, prostate cancer, lung cancer, melanoma and non-Hodgkin lymphomas [22–27]. It has been previously shown that heterozygous deletions at 1p36 were also found in 8% of MM patients, indicating that this region may contain putative tumor suppressor genes [28]. We analyzed published aCGH data from 238 MM patients and 60 human myeloma cell lines and found that about 8% MM patients have monoallelic deletion of 1p, which is consistent with the previous reports. The DNA methylation-mediated loss of GITR in MM may also provide evidence of alternative contribution of 1p36 abnormality to MM tumorigenesis.

Recent studies in Chronic Lymphocytic Leukemia (CLL) have shown pronounced expression of GITRL and the GITR receptor was expressed at significantly higher levels on NK cells of CLL patients compared with healthy controls. In addition, GITRL contributed to resistance of CLL cells to rituximab therapy, indicating that GITR/GITRL contributes to disease progression and resistance to Rituximab-induced NK reactivity in CLL [29]. In another study, constitutive expression of GITRL by tumor cells diminished NK cell antitumor immunity [30]. Moreover, in GITR-/- mouse models, the lack of expression of GITR was shown to modestly contribute to mature B cell homeostasis [31]. Our study demonstrated that the tumor suppressor properties of GITR are characterized by inhibition of proliferation and induction of apoptosis as well as induction of downstream targets of p53, such as p21, MDM2, Fas, IGF-BP3 and CD82. This data support the presence of a putative crosstalk between GITR and the p53 pathway. Therefore we attempted to elucidate the molecular basis that link p53 signal to the GITR pathway. We observed significantly increased p53 level in GITR+, leading to induction of p21 and puma in MM cells in response to GITRL. Further studies are needed to better define the interaction between GITR and the p53-related signaling cascade.

**Figure 6:**
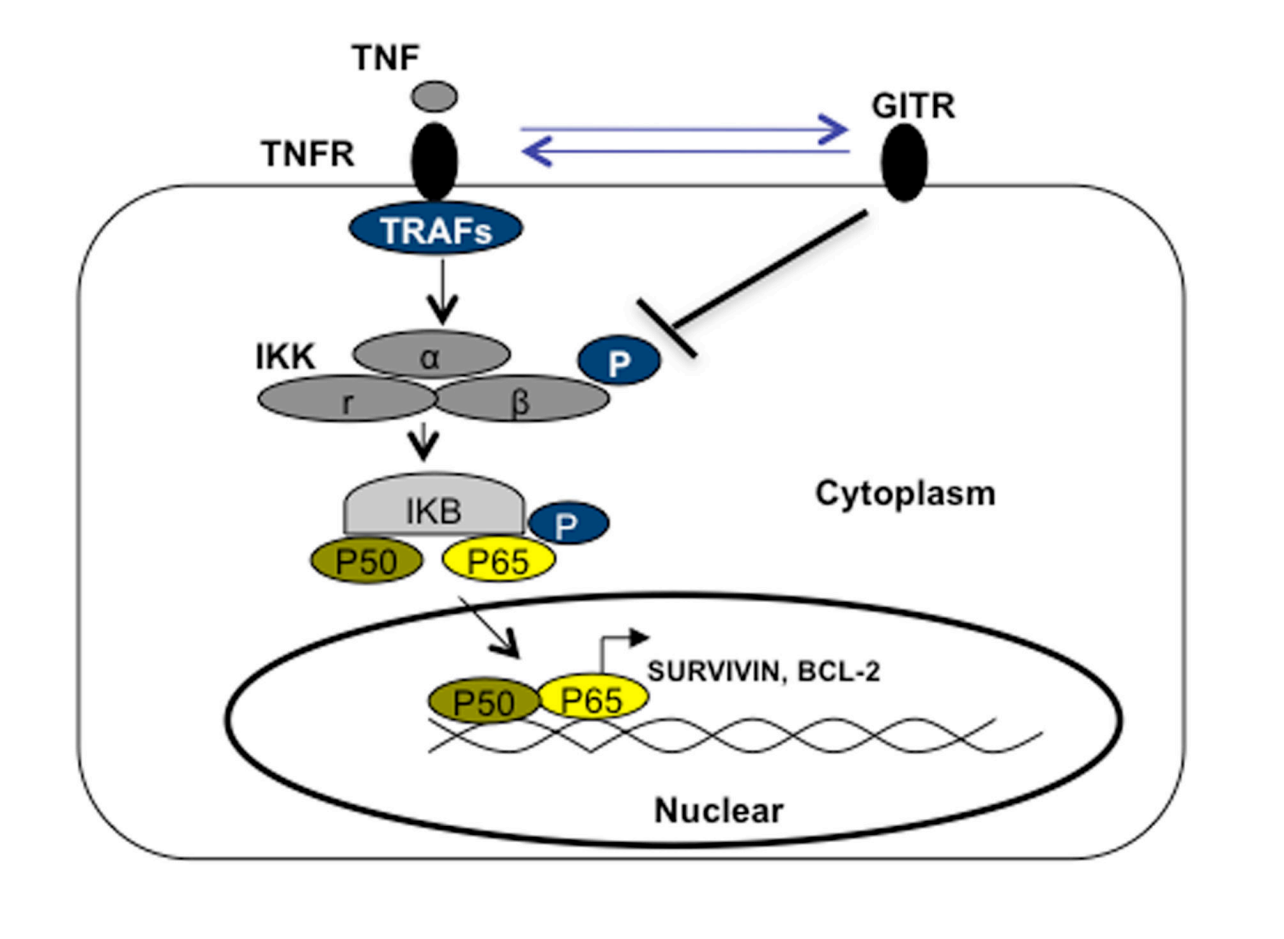
GITR inhibits NF-κB pathway. A proposed model to summarize the ability of GITR to modulate TNF-α induced canonical NF-κB pathway. The presence of GITR in MM cells could disrupt TNF-α/TNFR induced NF-κB activation by blocking phosphorylation of IKK-beta, subsequenctly leading to early termination of nuclear translocation of NF-κB p65/p50 and impaired induction of BCL-2 and Survivin.

NF-κB transcription factors play a key role in the survival and proliferation of plasma cell malignancies, including multiple myeloma [32–34]. It was shown that mutations involved in the NF-κB pathway are present in at least 17% of MM tumors and 40% of MM cell lines [35–37]. These mutations can lead to activation of the canonical NF-κB pathway via modulating the level of NIK, TRAFs and CD40. Therefore, targeting the NF-κB pathway is an attractive therapy for MM [20,38]. Our data indicates that GITR negatively regulates the canonical NF-κB pathway by terminating the canonical NF-κB activation as early as 30 minutes post-stimulation with TNF-α. The inhibitory role of GITR in the NF-κB pathway was further supported by inhibition of phosphorylation of IKK-β and protection of degradation of IκB. We speculate that this effect could possibly result from either binding of TNF-α to GITR or blocking the intracellular cell signaling transduction of TNF-α by GITR (Figure 6). Further elucidation of GITR mediated disruption of the NF-κB pathway will be critical for understanding the role of GITR in the pathogenesis of MM and the development of future therapeutic agents.

Overall, our results demonstrate that GITR acts as a potential tumor suppressor in MM. Furthermore, our data links GITR to p53-associated modulation of p21 and puma in myeloma cells. Most importantly, our findings reveal an inhibitory role of GITR in regulating NF-κB pathway, providing a novel insight into the role of TNFRSFs family in MM pathogenesis and disease progression, thus supporting the therapeutic use of NF-κB inhibitors in MM patients who present with increased GITR methylation.

## Supporting Information

Figure S1 Deregulation of TNFRSFS in multiple myeloma. **A**) Expression of TNFRSFs member was assessed by analyzing GEO dataset GSE2658. Expression value was used to calculate the p-value by T-test. The heat map was generated for all of the TNFRSFs members. Significance of differential expression of TNFRSFs members, which were selected according to the p-value(<0.05), is shown by the intensity of red (up-regulation) versus blue (down-regulation).

Figure S2 Hypermethylation of GITR in MM cells. A) Loss of methylation enrichment of GITR promoter in the presence of demethylation agent 5’ azacytidine in MM cell line by meDIP assay. Fold enrichment was normalized to GAPDH. B) Methylation specific PCR was performed to evaluated the methylation status of primers MM cells from 5 patients. According to the BSP results, MM1.S were considered as positive control and ddH_2_O as negative control. C) DNA sequencing was done in primary MM DNA samples after bisulfate convertion followed by subcloning into T-easy vector. C > T represents unmethylated C, whereas C>C represents methylated C. D) expression of GITR mRNA level was examined by real-time PCR in the presence of 5’ azacytidine (5 µM) for 4 days. Total RNA was extracted and reverse transcripted with oligo_dT from INA6, U266 and RPMI8226 cell lines. The mRNA level of GITR was normalized to 18s. Mean ±SD.

Figure S3 Promoter DNA methylation leads to GITR silencing in MM cells. Re-expression of GITR protein level was determined by flow cytometry exposed to 5’ azacytidine in a dose depedent manner. Cells were harvested after 96 hrs of incubation with 5’ azacytidine and stained with anti-GITR-PE labeled antibody.

Figure S4 Expression of GITR affects MM cell proliferation. A) MM.1S cells have been transfected with either empty vector (contrl) or GITR (GITR+). Expression of GITR has been evaluated by flow cytometry. Anti-GITR-PE conjugated antibody has been used. B) Knockdown of GITR in RPMI8226 cell lines by siRNA was measured by real-time PCR. mRNA level was normalized to 18s. C) MM cells have been transfected with either empty vector (contrl) or GITR (GITR+) and cultured in presence or absence of HUVECs for 48 hours. Cell proliferation has been evaluated by BrdU assay. Mean ±SD.

Figure S5 Overexpression of GITR induced the expression of p21 and puma in a ligand dependent way. F) MM1.S cells were transfected with both GITR overexpressing vector (GITR+) and empty control vector (GITR-) respectively. RPMI8226 cells were transfected with si-scramble and pooled si-GITR oligo. Total RNA was extracted in 24 hours after the transfection. Expression of p21, TRADD, Fas and DAP has been evaluated by qRT-PCR, with normalization to 18s. Mean ±SD. G) MM cells were transfected with either empty vector (contrl) or GITR (GITR+). Total RNA was extracted in 12 hours after treatment with GITRL (5-10ng/mL). Expression of p21 and puma was evaluated by qRT-PCR, with normalization to 18s. Mean ±SD.

Table S1 Overxpression of GITR is associated with p53 pathway. Genes involved in p53 pathway are shown (cut off = 2 fold, p<0.05).
