# Supplementary figures and images for: Novel Tumor Suppressor Function of Glucocorticoid-Induced TNF Receptor GITR in Multiple Myeloma
Source: PLoS One. 2013 Jun 13;8(6):e66982. doi: 10.1371/journal.pone.0066982 (PMC3681775; doi:10.1371/journal.pone.0066982)

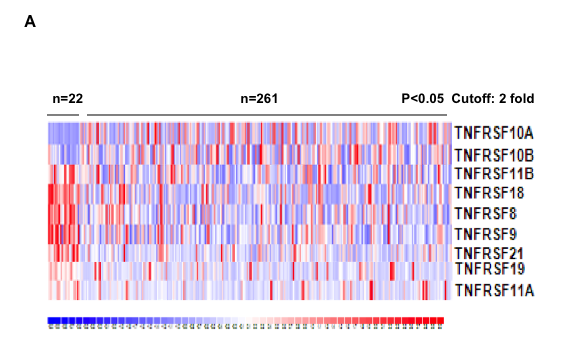

Supplement: Figure S1 — A) Expression of TNFRSFs member was assessed by analyzing GEO dataset GSE2658. Expression value was used to calculate the p-value by T-test. The heat map was generated for all of the TNFRSFs members. Significance of differential expression of TNFRSFs members, which were selected according to the p-value(<0.05), is shown by the intensity of red (up-regulation) versus blue (down-regulation). [file pone.0066982.s001.tiff]

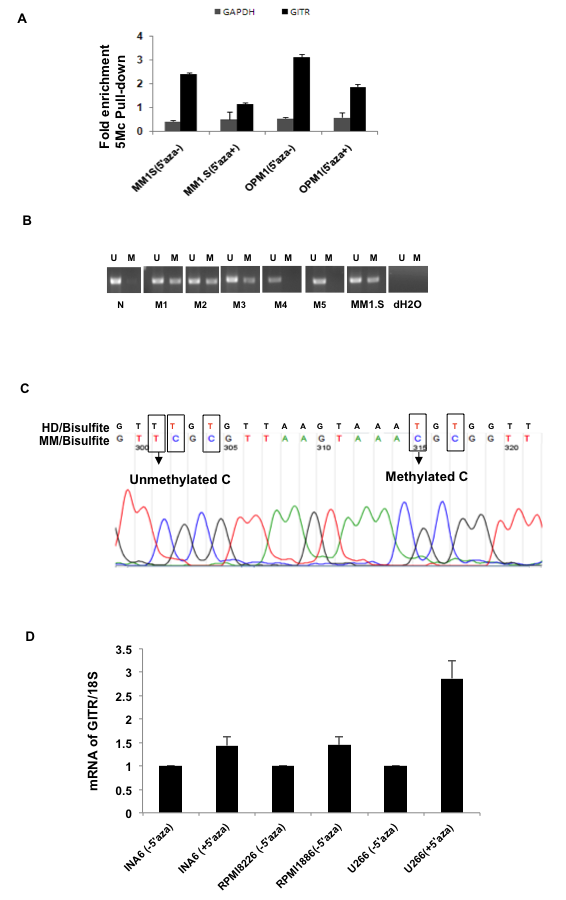

Supplement: Figure S2 — A) Loss of methylation enrichment of GITR promoter in the presence of demethylation agent 5’ azacytidine in MM cell line by meDIP assay. Fold enrichment was normalized to GAPDH. B) Methylation specific PCR was performed to evaluated the methylation status of primers MM cells from 5 patients. According to the BSP results, MM1.S were considered as positive control and ddH2O as negative control. C) DNA sequencing was done in primary MM DNA samples after bisulfate convertion followed by subcloning into T-easy vector. C > T represents unmethylated C, whereas C>C represents methylated C. D) expression of GITR mRNA level was examined by real-time PCR in the presence of 5’ azacytidine (5 µM) for 4 days. Total RNA was extracted and reverse transcripted with oligo_dT from INA6, U266 and RPMI8226 cell lines. The mRNA level of GITR was normalized to 18s. Mean ±SD. [file pone.0066982.s002.tiff]

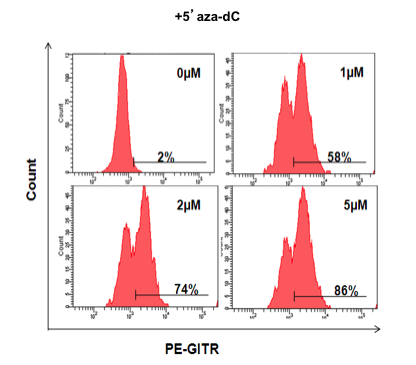

Supplement: Figure S3 — Re-expression of GITR protein level was determined by flow cytometry exposed to 5’ azacytidine in a dose depedent manner. Cells were harvested after 96 hrs of incubation with 5’ azacytidine and stained with anti-GITR-PE labeled antibody. [file pone.0066982.s003.tiff]

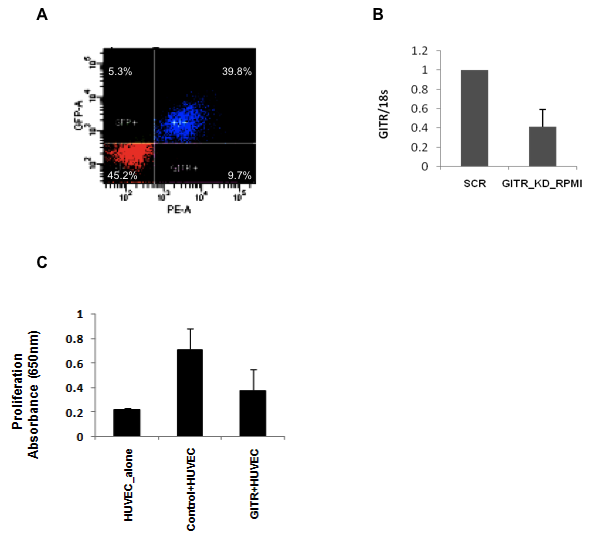

Supplement: Figure S4 — A) MM.1S cells have been transfected with either empty vector (contrl) or GITR (GITR+). Expression of GITR has been evaluated by flow cytometry. Anti-GITR-PE conjugated antibody has been used. B) Knockdown of GITR in RPMI8226 cell lines by siRNA was measured by real-time PCR. mRNA level was normalized to 18s. C) MM cells have been transfected with either empty vector (contrl) or GITR (GITR+) and cultured in presence or absence of HUVECs for 48 hours. Cell proliferation has been evaluated by BrdU assay. Mean ±SD. [file pone.0066982.s004.tiff]

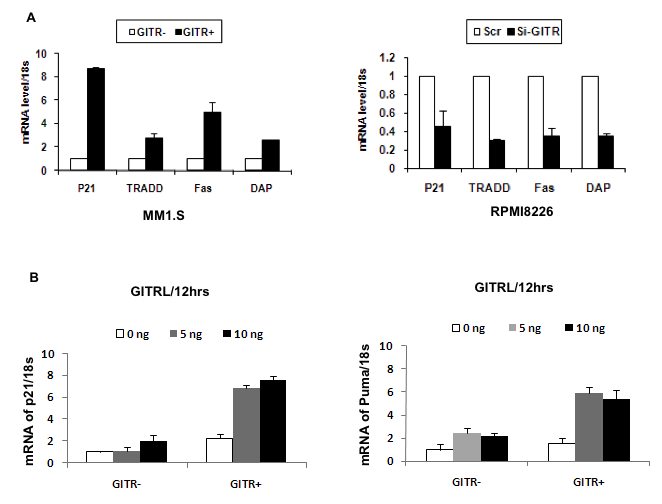

Supplement: Figure S5 — F) MM1.S cells were transfected with both GITR overexpressing vector (GITR+) and empty control vector (GITR-) respectively. RPMI8226 cells were transfected with si-scramble and pooled si-GITR oligo. Total RNA was extracted in 24 hours after the transfection. Expression of p21, TRADD, Fas and DAP has been evaluated by qRT-PCR, with normalization to 18s. Mean ±SD. G) MM cells were transfected with either empty vector (contrl) or GITR (GITR+). Total RNA was extracted in 12 hours after treatment with GITRL (5-10ng/mL). Expression of p21 and puma was evaluated by qRT-PCR, with normalization to 18s. Mean ±SD. [file pone.0066982.s005.tiff]

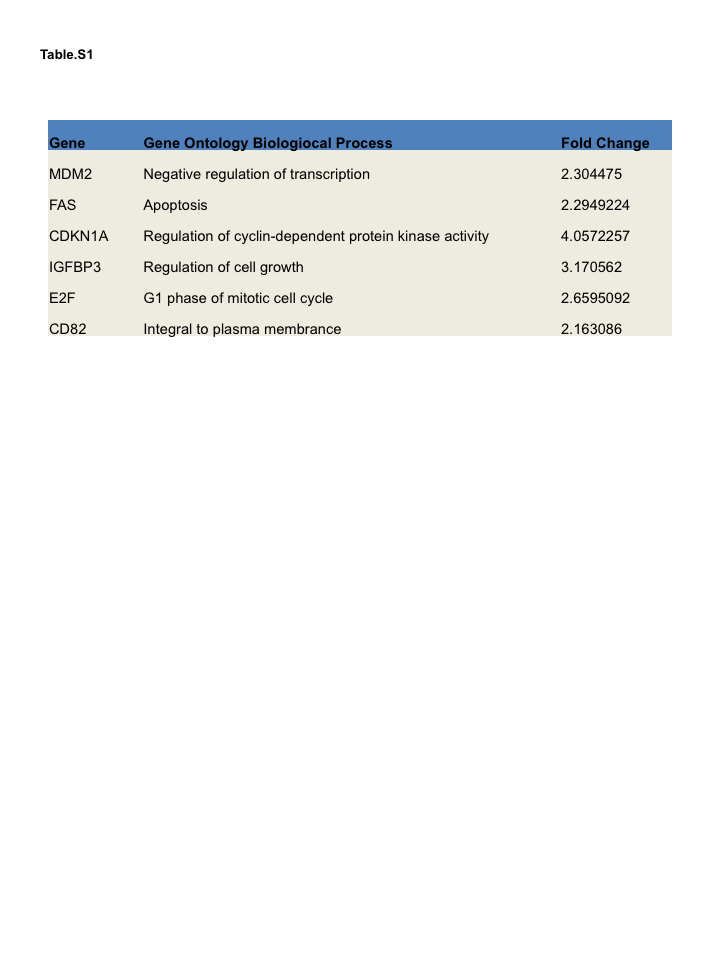

Supplement: Table S1 — Genes involved in p53 pathway are shown (cut off = 2 fold, p<0.05). [file pone.0066982.s006.tiff]
